# Does Japanese encephalitis virus share the same cellular receptor with other mosquito-borne flaviviruses on the C6/36 mosquito cells?

**DOI:** 10.1186/1743-422X-4-83

**Published:** 2007-09-06

**Authors:** Junping Ren, Tianbing Ding, Wei Zhang, Jianhua Song, Wenyu Ma

**Affiliations:** 1 Department of Microbiology, Fourth Military Medical University, 17 Changle West Road, Xi'an, 710032, People's Republic of China

## Abstract

Japanese encephalitis virus (JEV) is a member of mosquito-borne *Flaviviridae*. To date, the mechanisms of the early events of JEV infection remain poorly understood, and the cellular receptors are unidentified. There are evidences that the structure of the virus attachment proteins (VAP), envelope glycoprotein of mosquito-borne flaviviruses is very similar, and the vector-virus interaction of mosquito-borne flaviviruses is also very similar. Based on the studies previously demonstrated that the similar molecules present on the mosquito cells involved in the uptake process of JEV, West Nile virus (WNV) and Dengue virus (DV), it is proposed that the same receptor molecules for mosquito-borne flaviviruses (JEV, WNV and DV) may present on the surface of C6/36 mosquito cells. By co-immunoprecipitation assay, we investigated a 74-KDa protein on the C6/36 cells binds JEV, and the mass spectrometry results indicated it may be heat shock cognate protein 70(HSC70) from *Aedes aegypti*. Based upon some other viruses use of heat shock protein 70 (HSP70) family proteins as cell receptors, its possible HSC70's involvement in the fusion of the JEV E protein with the C6/36 cells membrane, and known form of cation channels in the interaction of HSC70 with the lipid bilayer, it will further be proposed that HSC70 as a penetration receptor mediates JEV entry into C6/36 cells.

## 1 Background

Japanese Encephalitis Virus (JEV) is an enveloped positive single stranded RNA virus belonging to genus *Flavivirus *in the family *Flaviviridae*. It is the most common agent of viral encephalitis, causing an estimated 50,000 cases annually, of which 15,000 will die and up to 50% of survivors are left with severe residual neurological complications [1,2]. Most cases occur in southern and eastern Asia, but the geographical area affected by JEV is expanding. Outbreaks have been reported in Saipan islands, Torres Straits islands and on Australia mainland in recent years [3-5]. Cases have also occurred among travelers and US servicemen to Asia [6,7].

The first step in virus infection requires interaction between the virus attachment proteins (VAP) and cellular receptors. The interaction of VAP and its cellular receptors is known to contribute to host range, tissue tropism and viral pathogenesis. The characteristic and function of virus receptor, once ascertained, may ultimately lead to the production of effective antiviral agents. But what are the cellular receptors for JEV? How the cellular receptors for JEV mediate JEV entry into the host cells?

To look for the answers to these questions, the authors made a detailed analysis on the JEV receptors, based upon previous flaviviruses receptor research results on Dengue virus (DV) and West Nile virus (WNV). By analyzing and summarizing the known characteristics of flaviviruses receptors on mosquito cells, we raised a hypothesis stating that a 74-KDa heat shock cognate protein 70(HSC70) may act as a penetration receptor for JEV on mosquito cells.

## 2 Discussion

### 2.1 The same receptor molecule(s) for mosquito-borne flaviviruses (JEV, WNV and DV) might present on the surface of mosquito cells

The classic notion of a virus binding to a single receptor to enter cells is being overtaken by the more complex conception. One of the complex concepts is that a virus particle can use multiple (individual) receptors during cell entry. Several viruses use at least two different receptors to interact with their host cells: (i) the binding receptors, which in general allow the virus particle to rapidly attach to the cell surface, and (ii) receptors that are used by the virus after binding to the cell, which are referred to using different terminology, such as post-binding, post-attachment, entry, fusion, internalization, secondary or co-receptors, depending on the function that they are known or proposed to play during the process of virus infection. In some instances, such as in the case of human immunodeficiency virus-1 (HIV-1), herpes simplex virus 1 and 2, adenovirus and measles virus [8-12], the multiple interactions that take place between the virus and cell surface molecules have been proposed to occur in a sequential manner. A prominent example of a dual receptor requirement occurs with HIV-1 binding. HIV-1 uses CD4 to bind the cell surface and chemokine so-called co-receptors, such as CXCR4 and CCR5, to facilitate the conformational alterations in envelope glycoproteins that culminate in fusion of the viral envelope and cell membrane.

Another complex concept is that very different viruses may use identical receptors. Although in most cases individual viruses have their own distinct receptors, in some cases the same receptor can be used by quite different viruses. Perhaps the best studied example of this is the coxsackie-adenovirus receptor or CAR [13]. CAR is a member of the immunoglobulin superfamily and mediates both attachment and entry of these two viruses. Another example is the integrins, that have been identified as attachment and entry receptors for several viruses[14], including reovirus (β1 integrins), echovirus (α2β1), foot-and-mouth disease virus (αvβ1, αvβ3, and αvβ6), hantaviruses NY-1 and Sin Nombre virus (β3 integrins), Kaposi sarcoma herpesvirus (α3β1), rotavirus (α2β1, αvβ3, αxβ2, and α4β3) and cytomegalovirus (α2β1, α6β1, and αvβ3). Integrins are a family of cell surface receptors that consist of α and β subunits. Integrins mediate cellular adhesion to the extracellular matrix (ECM), regulate cellular trafficking, and transducer both outside-in and inside-out signaling events.

**Figure 1:**
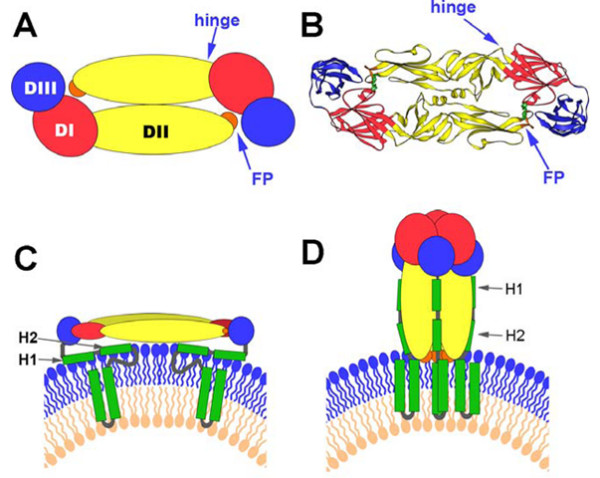
Summary of the Structural Organization and Different Conformations of the Flavivirus Envelope Protein E (obtained the kind permission from the copyright holder to reproduce figures that have previously been published on [51]). (A) Schematic top view of the organization of the sE protein dimer as present at the surface of mature virions, color-coded according to the three domains (DI, DII, and DIII). The fusion peptide (FP) is indicated in orange. (B) Crystal structure (top view) of the TBEV E ectodomain (termed "sE") dimer. (C) Schematic side view of the DV E dimer at the surface of mature virions, with the "stem" and TM C-terminal polypeptide segments (missing in the truncated sE form) indicated in green. The viral lipid bilayer is illustrated with lipids belonging to the outer and inner leaflets colored blue and pink, respectively. Cryo-electron microscopy 3D reconstructions have shown that the stem forms two α-helices (H1 and H2) lying on the viral membrane, followed by the two  transmembrane (TM) segments. (D) Schematic representation illustrating the proposed organization of full-length DV E in its postfusion conformation. In this model, the α-helices of the stem interact with the body of the trimer, in the grooves between adjacent, parallel DIIs. The lipid bilayer as well as the stem and TM segments is drawn as in (C).

Flaviviruses package their positive-strand RNA genome into particles consisting of a rigid outer protein shell and an underlying lipid membrane. The major envelope glycoprotein, E, and a small membrane protein, M, form the outer shell. As the principal envelope component, E is responsible for receptor binding and membrane fusion. Flavivirus E proteins belong to the structurally conserved "class II" fusion proteins, which are also found in alphaviruses. Crystal structures of four class II fusion proteins – Tick-borne encephalitis virus (TBEV) E [15,16], Dengue virus (DV) E [17-19], Semliki Forest virus (SFV) E1 [20,21], and West Nile virus (WNV) E [22,23] – before and after their fusogenic conformational rearrangements provide us with a detailed molecular picture of the fusion mechanism of these viruses. Based on the work of the above, it has been demonstrated that the E protein of the mature flaviviruses forms homodimers in an anti-parallel manner (head-to-tail orientation). Each monomer is folded into three distinct domains (Fig 1A and 1B), namely domain I (DI) – the central N-terminal domain; domain II (DII) – the dimerization domain; and domain III (DIII) – the immunoglobulin (Ig)-like domain. DIII of E protein consists of 100 amino acids (residues 303–395) of the C-terminal. This domain has been suggested to be the receptor recognition and binding domain due to a number of reasons. The Ig-like fold present in the DIII protein is commonly associated with structures that have an adhesion function. This domain extends perpendicularly to the surface of the virus with a tip projecting further from the virion surface than any part of the E protein. In addition, studies have demonstrated that both recombinant DIII proteins and antibodies generated against DIII of E protein of flaviviruses can inhibit entry of the flaviviruses into target cells [24-26]. In addition, flaviviruses with mutations in DIII of the E protein show either attenuated virulence or the ability to escape immune neutralization [27-30].

Neuroinvasiveness is a common feature of mosquito-borne flaviviruses infections where *Culex *mosquitoes are the predominant mosquito vector. In natural infection, mosquito-borne flaviviruses are first deposited in the mosquito vector and then in a human host bitten by the vector during a blood meal. Therefore, it is necessary to study receptors in mosquito cells to determine which binding proteins serve as true virus receptors. Several studies on the mosquito-borne flaviviruses, DV [31-36], WNV [37], have been made significant progress in identifying a number of putative cellular receptors [glycolsaminoglycans, DC-SIGN, laminin receptor, glucose regulated protein 78 (GRP78), heat shock protein 90 (HSP90) and heat shock protein 70 (HSP70), αVβ3 integrin] in different mammalian cell types. However, the search for cellular receptor in mosquito cells has been less successful. A series of protein bands of different molecular masses on the mosquito cells (C6/36 cells) or mosquito tissues were observed to bind to DV [38-42], WNV [43] and JEV [44].

Two glycoproteins of apparent molecular weights of 40- and 45-kDa expressed on the surface of C6/36 cells and mosquito tissues have been previously identified by del Angel and colleagues [38-40] as potential Dengue virus serotype 4 (DV-4) receptor proteins. The 45-kDa protein has been shown to be widely expressed in mosquito tissues and antibodies against this protein specifically inhibit DV-4 infection. They demonstrated that periodate treatment of C6/36 cells protein extract modified the molecular weight of the 40- and 45-kDa glycoproteins to a 38-kDa protein, which was also able to bind DV-4. Therefore, the 40- and 45-kDa molecules are probably the glycosylated forms of the 38-kDa molecule. Sakoonwatanyoo and colleagues [41] identified a laminin-binding protein of 50-kDa on the surface of C6/36 cells as a candidate DV 2, 3, and 4 receptor protein. The authors have noticed that the marker used in their study gives higher calculated molecular weights than other markers. As such, they thought that the protein of 50-kDa identified by them and the 45-kDa protein identified by del Angel *et al *are probably the same protein. Another two proteins with molecular masses of 80- and 67-kDa on the midgut cells of *Aedes aegypti and C6/36 cells *have been recently identified as receptors for the four serotypes of DV [42]. Interestingly, Chu *et al *[43] identified proteins (55-kDa, 70-kDa, 95-kDa and 140-kDa) implicated in the binding and internalization of WNV, of which two (70- and 95-kDa) were proposed to be part of the receptor complex for mosquito-borne flaviviruses (WNV, JEV and DV) on C6/36 cells, as antibodies against these proteins blocked WNV, JEV and DV-2 entry into C6/36 cells. More recently, Boonsanay *et al *[44] identified several proteins on the surface of C6/36 cells binding to JEV, and predominant band was noted at 53-kDa, while other bands in the range of 150 to 200-kDa and minor bands of approximately 35-, 51- and 80-kDa were also observed. Soluble laminin produced a marginal, but dose-dependent inhibition of JEV infection, suggesting laminin role in virus-receptor interaction.

Collectively, on the surface of C6/36 cells or mosquito tissues the molecular masses approximately 38-, 40-, 45-, 50-kDa proteins have been identified as a candidate DV receptor protein, and are probably the same protein, laminin-binding protein. Another two proteins, molecular masses approximately 67 to 70-kDa and 80 to 95-kDa are probably a part of the receptor complex for mosquito-borne flaviviruses (JEV, WNV and DV). The evidences are listed as following: (i) the structure of VAP, envelope glycoprotein of mosquito-borne flaviviruses is very similar described as above; (ii) the vector-virus interaction of mosquito-borne flaviviruses is very similar. Based on the studies previously demonstrated that the similar molecules present on the mosquito cells involved in the uptake process of JEV, WNV and DV, it is proposed that the same receptor molecules for mosquito-borne flaviviruses (JEV, WNV and DV) may present on the surface of mosquito cells.

### 2.2 Heat shock cognate protein 70 (HSC70) may be a penetration receptor mediate JEV entry into C6/36 cells

To date, there has been little progress in identifying the nature of the molecules involved in the initial JEV entry, except that one report previously identified a 74-kDa protein expressed by Vero cells that may be involved in the uptake process [45], and another report identified several proteins on the surface of C6/36 cells binding to JEV [44]. However, the detailed characteristics and functioning of JEV receptor still remain a mystery.

We tried to investigate those proteins binding to JEV on the surface of C6/36 cells. Several positive proteins were observed in co-immunoprecipitation assay, and corresponding bands were then subject to mass spectrometry (data not published so far). The results indicated only a 74-kDa band may be HSC70 from *Aedes aegypti*. Amazingly, after transferred to nitrocellulose membrane, the 74-kDa protein from C6/36 cells was shown to bind only with HSC70 antibody but not with HSP70 antibody. Thus, we preferred that the 74-kDa HSC70 may be a cellular receptor for JEV. Further experiments directed to confirm the 74-KDa protein's activity as a putative cellular receptor for JEV are being performed in our laboratory. Such judgment we made is reasonably deducible due to the following reasons.

First, some other viruses, including human T lymphotropic virus type 1 (HTLV-1) [46], coxsackievirus A9 [47], rotavirus [48] and Dengue virus [35,36] have been reported to use HSP70 family proteins as cell receptors. Specifically, GRP78 and HSP70 have been described as a member of a receptor complex on the mammalian cells for mosquito-borne flavivirus, Dengue virus. The HSP70 family is composed of four highly conserved proteins: HSP70, HSC70, GRP75 and GRP78. These proteins serve a variety of roles, such as acting as molecular chaperones facilitating the assembly of multi-protein complexes, participating in the translocation of polypeptides across cell membranes and to the nucleus, and aiding in the proper folding of nascent polypeptide chains. Virus proliferation depends on the successful recruitment of host cellular components for their own replication, protein synthesis, and virion assembly. HSP70 chaperones, as central component of the cellular chaperone network, are frequently recruited by viruses. Although HSP70 and HSC70 do not contain export signal peptide sequences, and more importantly depend in their chaperone function on repetitive cycles of ATP hydrolysis, they are found on the cell surface of a number of different cell types including tumor cells, virus infected cells, spermatogenic cells, epidermal cells, arterial smooth muscle cells, monocytes and B cells [49].

Second, HSC70 as chaperones might participate in the conformational changes of JEV envelope glycoprotein membrane fusion. Flaviviruses enter cells by receptor-mediate endocytosis, and the acidic pH in the endosome triggers the fusion of the viral envelope with the membrane of the target cells [50]. The crystal structures of the E ectodomain (termed "sE") were determined for four flaviviruses in both their prefusion and postfusion conformation [15-23]. Flaviruses use only a single envelope glycoprotein (E) to mediate the membrane fusion during virus entry. Stiasny *et al *[51] recently reported a model to explain the conformational changes in E protein membrane fusion. In the prefusion form, as shown in Figure 1A and 1B, the three domains of sE are aligned along a rod-like molecule, with the C terminus and the fusion peptide (FP) lying at the two distal ends of the molecule. In full-length E, the sE segment connects to the C-terminal transmembrane (TM) segments via an element of about 50 amino acids (called "stem") that contains two α-helices, H1 and H2, which are peripherally attached to the viral envelope (Figure 1C). In the postfusion form, as shown in Figure 1D and 1E is converted into a more stable trimeric conformation. The structure of sE in the trimer shows that DIII is displaced from its original location and thus becomes positioned at the side of DI with its C terminus pointing toward the FP. This domain is the one that undergoes the most significant displacement in the dimer-to-trimer transition. In this scenario, it is tempting to speculate that a protein with chaperone activity, like HSC70, could have a pivotal role to help in these processes. By binding JEV E protein through DIII, it appears that HSC70 not only serves as an anchor on the cell membrane, but also modifies the conformational changes of dimer-to-trimer. This idea is consistent with the known functions of the HSC70 protein. For this reason, it is rational to hypothesize that HSC70 as a chaperone might participate in this proposed transition of JEV E protein.

Finally, HSC70 as a penetration receptor may mediate JEV entry into the C6/36 cells. To infect, a virus must first attach itself to the surface of a susceptible cell. The molecules to which viruses bind constitute a diverse collection of cellular proteins, carbohydrates, or lipids. Some of them merely serve as attachment factors that concentrate viruses on the cell's surface. Others are true receptors in that they not only bind viruses but are also responsible for guiding the bound viruses into endocytic pathways and for transmitting signals to the cytoplasm. These receptors can also serve as cues that induce conformational changes leading to membrane fusion and viral penetration [52].

All members of the HSP70 family contain three structural and functional domains [49]. The domain at the N terminus of the molecule (44-kDa) binds and hydrolyzes ATP. The subsequent region (18-kDa) participates in the interaction with target proteins (peptide binding domain). The C terminus of the molecule (10-kDa) seems to be involved in the association with co-chaperone molecules such as DnaJ. The interaction of HSP70s with peptides is modulated by the presence and hydrolysis of ATP. Thus, ATP is necessary for the recognition of the peptide, whereas hydrolysis of ATP to ADP increases the affinity for the peptide. The interaction of HSP70s with membranes may be necessary for the translocation of polypeptides across these lipid barriers. HSC70 is also known to interact with lipids, and it has been shown that this protein is able to form cation channels in acidic phospholipid membranes [53]. The HSC70 channel activity is ATP-dependent and is reversibly blocked by ADP. This channel has cationic selectivity. Perhaps the interaction of HSP70 with lipids is important in the processes of translocation and folding of membrane proteins. In addition, hydrophobic patches of HSC70, which are thought to be likely regions for interaction with membrane lipids, have been observed in the C-terminal of the ATP binding domain [54] and in the N-terminal of the peptide binding site [55], which may be involved in the interaction with the lipid bilayer.

Therefore, based upon HSC70's possible involvement in the fusion of the JEV E protein with the C6/36 cell membrane as described above, and its known form of cation channels in the interaction of HSC70 with the lipid bilayer, it is reasonably proposed that HSC70 as a penetration receptor mediate JEV entry into C6/36 cells.

## 3 Conclusion

Based upon several lines of evidence, it is reasonable to infer that mosquito-borne flaviviruses may share the same receptor molecule(s) on mosquito cells, because these viruses must replicate in mosquito cells first before injecting into host animals, including human. Compared with other mosquito-borne flaviviruses, Dengue is not a typical neuron-invasive virus, while JEV and WNV are characteristic with similar genome, neuron-invasive, and biological properties. Yet, it is also notable that more and more case reports recently indicated a increasing of Dengue infection manifested as encephalitis with unknown mechanism [56,57]. However, the discrepancy of pathogenesis and clinical manifestations of these viruses strongly suggests that these viruses must have different receptor(s) and pathogenesis on human. Even so, identification and characterization of receptor on mosquito cells is the prologue to final elucidation of flavivirus pathogenesis on human. To confirm that HSC70 is a receptor for JEV on C6/36 mosquito cells, we are trying to do several experiments in our laboratory to: (i) make sure that JEV E protein interacts with HSC70 from C6/36 cell membranes by pull-down assay; (ii) test if antibodies against HSC70 block JEV infection, or inhibition of HSC70 expression by small RNA interference technique decrease JEV infection; and (iii) further define the specific interaction site(s) of HSC70 and JEV E protein.

Additionally, future research should define the role of HSC70 in JEV entry, identify any other co-receptors of JEV if existing, determine the route of JEV entry, and reveal the specific mechanism of JEV internalization. A combination of standard biochemical and molecular tools, together with the use of other technologies, such as RNA interference, as well as high-resolution structural cryo-electron microscopy and X-ray crystallography, will be required to gain insight into the elaborate mechanism employed by JEV to enter cells. The unveiled domain of JEV E protein in the conformational change interactions with cellular receptors could be a target of neutralizing antibodies or antiviral drugs. Such complexity pertaining to virus entry may make discovering treatments targeting this stage of infectious cycle more challenging, but the specificity involved in the processes, once ascertained, may ultimately lead to the production of effective antiviral agents or developments of new viral vaccines.

## Competing interests

The author(s) declare that they have no competing interests.

## Authors' contributions

JR and TD produced the ideas and drafted the manuscript. WZ and JS helped to comment on the manuscript. WM finalized the manuscript. All authors read and approved the final manuscript.
